# Parents’ Experiences of Communication in Neonatal Care (PEC): a neonatal survey refined for real-time parent feedback

**DOI:** 10.1136/archdischild-2022-324548

**Published:** 2023-01-30

**Authors:** Susanna Sakonidou, Sophia Kotzamanis, Amy Tallett, Alan J Poots, Neena Modi, Derek Bell, Chris Gale

**Affiliations:** 1 Neonatal Medicine, School of Public Health, Imperial College London, London, UK; 2 Picker Institute Europe, Oxford, UK; 3 NIHR CLAHRC for Northwest London, London, UK

**Keywords:** neonatology, intensive care units, neonatal, intensive care units, paediatrics, child health services

## Abstract

**Objective:**

Assessing parent experiences of neonatal services can help improve quality of care; however, there is no formally evaluated UK instrument available to assess this prospectively. Our objective was to refine an existing retrospective survey for ‘real-time’ feedback.

**Methods:**

Co-led by a parent representative, we recruited a convenience sample of parents of infants in a London tertiary neonatal unit. Our steering group selected questions from the existing retrospective 61-question Picker survey (2014), added and revised questions assessing communication and parent involvement. We established face validity, ensuring questions adequately captured the topic, conducted parent cognitive interviews to evaluate parental understanding of questions,and adapted the survey in three revision cycles. We evaluated survey performance.

**Results:**

The revised Parents’ Experiences of Communication in Neonatal Care (PEC) survey contains 28 questions (10 new) focusing on communication and parent involvement. We cognitively interviewed six parents, and 67 parents completed 197 PEC surveys in the survey performance evaluation. Missing entries exceeded 5% for nine questions; we removed one and format-adjusted the rest as they had performed well during cognitive testing. There was strong inter-item correlation between two question pairs; however, all were retained as they individually assessed important concepts.

**Conclusion:**

Revised from the original 61-question Picker survey, the 28-question PEC survey is the first UK instrument formally evaluated to assess parent experience while infants are still receiving neonatal care. Developed with parents, it focuses on communication and parent involvement, enabling continuous assessment and iterative improvement of family-centred interventions in neonatal care.

**WHAT IS ALREADY KNOWN ON THIS TOPIC**

- It is important to assess parents’ experiences of neonatal services to understand how quality of care can be improved continuously.
- There is inconsistency and lack of performance evaluation of instruments measuring parent satisfaction in neonatal care worldwide.
- To date, no UK survey has been formally evaluated for measuring parent satisfaction with neonatal care while infants are still inpatients.

**WHAT THIS STUDY ADDS**

- We report the development and refinement of a 28-question prospective parent experience survey, in collaboration with parents in neonatal care.
- The Parents’ Experiences of Communication in Neonatal Care (PEC) survey is the first survey in the UK formally evaluated to obtain ‘real-time’ parent feedback while infants are still receiving neonatal care.
- The PEC survey focuses on communication and parent involvement to enable continuous assessment and iterative improvement of family-centred interventions in neonatal care.

**HOW THIS STUDY MIGHT AFFECT RESEARCH, PRACTICE OR POLICY**

- This survey can be used to conduct one-off service evaluation of real-time parent experience on neonatal units and to inform the development of new interventions.
- It can be used for continuously assessing research, audit and quality improvement projects, supporting the iterative improvement of family-centred interventions with parents in neonatal care.

## Introduction

One in seven babies born in the UK and other high-income countries will receive hospital care on a neonatal unit.^1^ This is an understandably stressful time for parents, with as many as 35% reporting symptoms of anxiety, depression and post-traumatic stress disorder following neonatal care.^2–4^ Parental stress has been shown to interfere with parent–child bonding,^5^ and there is a well-established link between maternal mental health and infant development.^6^ Parent satisfaction, defined as ‘the perception of parents’ needs and expectations being met’ is inversely related to parental stress.^7^ Consequently, interventions aimed at improving parent satisfaction are being developed worldwide^8^ to reduce parent stress, improve parent–infant bonding^9^ and outcomes.^10 11^

In 2009, the UK Department of Health published the *Toolkit for High-Quality Neonatal Services*, which emphasised the importance of family-centred care.^12^ The UK national neonatal charity Bliss developed a ‘baby charter’ in 2011, which outlined components of high-quality family-centred care with reference to ‘a culture of continuous improvement that involves and is informed by parents’.^13^ To continually improve the quality of neonatal care, and family-centred care in particular, neonatal services must be able to assess parental experiences and parent satisfaction with neonatal services.

Robust measurement of parent satisfaction is challenging. Inconsistent and unvalidated instruments are commonly used. Most existing validated instruments in the English language worldwide have been primarily designed for retrospective parent feedback, near to or after discharge from neonatal care.^8 14^ In UK neonatal care, only one validated survey for parents in neonatal care exists: the Parents’ Experiences of Neonatal Care. This survey was developed in 2010 by Picker Institute Europe (Picker) and revised in 2014, in collaboration with Bliss and UK neonatal network representatives to measure parent satisfaction after a baby had been discharged from neonatal care. It has been successfully used to measure and understand experiences of neonatal care in England.^15 16^ However, there are no formally evaluated instruments in the UK that prospectively measure parent satisfaction within or for neonatal care. Interventions being developed to improve parent satisfaction^8^ require a formally evaluated and robust prospective measure of parent satisfaction.

Our aims were

- To refine the Parents’ Experiences of Neonatal Care survey to measure the satisfaction of current parents in neonatal care prospectively. This prospective instrument would provide ‘real-time’ feedback to enable continuous ‘measurement for change’ in neonatal research, audit and quality improvement projects.
- To focus the new survey on measuring parent satisfaction specifically with ‘communication of clinical information’ and ‘involvement in care’, to facilitate more specific evaluations of family-centred interventions in neonatal care in the UK.
- To evaluate the performance of the revised survey.

## Methods

This study was co-led by a parent representative in two stages.

### Stage 1: survey design and revision

Picker licensed the Parents’ Experiences of Neonatal Care survey to Imperial College London for adaptation. The survey included 61 questions in 10 sections, covering all aspects of parent experience, in the past tense. The 10-member steering group included clinical experts (neonatologists and neonatal nurses), two neonatal parent representatives, a Bliss neonatal charity representative and methodology experts from Picker (full details in online supplemental file 1). The steering group selected questions from the survey that focused on assessing parent satisfaction with communication and parent involvement, added new questions for further exploration of these topics (as primarily suggested by the parent representatives, supported and advised by the neonatal charity representative) and modified all question wording to capture information prospectively.

10.1136/fetalneonatal-2022-324548.supp1 Supplementary data

### Stage 2: survey evaluation

We conducted our evaluation of survey performance over three phases:

- Face validity. The steering group evaluated the survey to ensure the questions adequately captured the topic under investigation and experts on survey construction checked the survey for ‘common errors’, such as double-barrelled, confusing or leading questions. Final questions were approved by the parent representative as appropriately capturing the topic using parent-friendly language.
- Cognitive testing. Interviews with six parents of babies that were inpatients on a single UK tertiary National Health Service (NHS) neonatal unit were undertaken to evaluate how adapted and new questions performed. Parents aged 16 or older who could speak English were randomly selected when their baby was in the low-dependency neonatal unit area. Because the Parents’ Experiences of Neonatal Care Picker survey had been extensively validated and adjustments to selected survey questions were expected to be minor, six parents were estimated to be sufficient for testing the revised survey. Parents provided written informed consent. The lead researcher undertook a 30 min cognitive interview with each parent in a neonatal unit private room; this explored the parents’ question interpretation and response processes, using the model described by Tourangeau.^17^ The researcher asked parents to answer the survey’s questions while thinking aloud, using techniques including probing, paraphrasing and observation. This explored parents’ thought processes to ensure consistency in how they understood the questions and drew on their experiences to answer appropriately. The survey was adapted through three revision cycles, including wording and formatting changes in response to parent feedback during interviews.
- Data evaluation. We distributed the revised survey to all parents on the neonatal unit two times a week for 3 months to evaluate the performance of individual survey questions. We evaluated the following domains:
  - Response completion rates. If any question had more than 5% missing responses, this was considered for removal.
  - Dropout. We identified the last question answered by each parent. If there were many people dropping out at a particular question, then it might indicate there was a problem with the question or the survey was too long.
  - Uninformative responses. If the percentage of ‘I don’t know’ or not applicable (‘N/A’) responses was more than 5%, the question was considered for removal.
  - Differentiation. We assessed data for hugely negative or positive (floor and ceiling) responses, defined as >95% negative or positive.
  - Inter-item correlation. We used Kendall’s tau to check for inter-item correlation of survey questions with numerical answers.^18^ A correlation coefficient of >0.7 or <−0.7 signified a large correlation. If questions met this threshold, we assessed them side-by-side to understand if both were necessary to retain.

Analysis was undertaken using SPSS V.25.

## Results

### Stage 1: survey design and revision

The study steering group selected 18/61 questions from the original survey, omitting questions not relevant to communication with parents or involvement in care, and added 9 new questions. All questions were modified to enable prospective measurement; for example, ‘If you ask(ed) questions about your baby’s condition and treatment (became (ask)), d(id) you get answers you could understand (became (do))’. The survey was renamed ‘The PEC questionnaire: Parents’ Experiences of Communication in Neonatal Care’.

### Stage 2: survey evaluation

#### Face validity

The steering group discussed all questions over the course of a 2 hour meeting and, with SS mediating any disagreements, determined a set of questions on which the group agreed.

#### Cognitive testing

Ten parents were approached between 1 August 2018 and 12 September 2018, of which six were recruited (five mothers and one father). The infants of 2 of 10 parents were discharged before parents consented; 1 parent was excluded because their English understanding was not deemed sufficient (this occurred before recruitment but after initial screening); and 1 parent did not visit the unit again to be recruited. Table 1 shows demographic details for parents who were interviewed. Four out of six parents were from a white ethnic background, and most were 30–35 years of age. Infant gestations at birth ranged 23–37 weeks (most under 30 weeks), and length of stay ranged 2 weeks–5 months (most 2–4 weeks). Following parent interviews and one revision cycle after every two interviews, we made changes to the administered Parents’ Experiences of Communication in Neonatal Care (PEC) survey questions, including wording and formatting, (eg. questions presented in subsections, N/A options given), and added a further new question for further exploration and depth (online supplemental file 2).

10.1136/fetalneonatal-2022-324548.supp2 Supplementary data

**Table 1:** Demographic information of parents in cognitive interviews (n=6)

Gender (n)	Age (years) (n)	Ethnicity (n)	Gestation (weeks)	Length of stay (n)
Female (5)	30–35 (4)	White (4)	<24 (one infant)	2–4 weeks (4)
Male (1)	25–29 (2)	Black (1)	24–28 (two infants)	1–2 months (1)
		Indian (1)	28+1–32 (two infants)	4–6 months (1)
			36+1–40 (one infant)	

#### Data evaluation

The final PEC survey is available in online supplemental file 4. Sixty-seven parents of babies receiving neonatal care completed 197 PEC surveys over 3 months (some parents completed the survey more than once, each time with regard to their experience of neonatal care at the point in time in which they were given a survey). Survey completion times by parent ranged between 1 and 12 times (mean 2.42), with 28 parents being one-time responders. Most respondents were female, aged over 30, and white (table 2). Demographic details for five parents were missing.

10.1136/fetalneonatal-2022-324548.supp4 Supplementary data

**Table 2:** Demographic information of survey responses (N=192)

Gender (%)	Age (years) (%)	Ethnicity (%)	Gestation (weeks) (%)	Length of stay (%)
Female (77)	Over 35 (39)	White (53)	<24 (3)	<1 week (20)
Male (23)	30–35 (36)	Mixed (5)	24–28 (31)	1–2 weeks (14)
	25–29 (17)	Asian/Asian British (23)	28+1–32 (38)	2–4 weeks (30)
	18–24 (8)	Black/black British (10)	32+1–36 (15)	1–2 months (26)
		Other (9)	36+1–40 (12)	2–4 months (8)
			>40 (1)	4–6 months (1)
				>6 months (1)

- Response completion rates. Nine questions had >5% non-response rates. We reformatted and retained eight of nine questions as they performed well during cognitive testing and removed one question. We include examples of reformatting for the three questions with the highest non-response rates (table 3).
- Dropout. Two per cent of parents dropped out prior to the penultimate question and 58% of parents after its penultimate question. As the last question was an open question, where non-responses are common, no changes were made to the survey.
- Uninformative responses. No questions exceeded uninformative responses of 5% or more.
- Differentiation. In testing for floor/ceiling responses, no question had >95% negative responses. Question A4 (‘Have you been told which nurses are responsible for your baby’s care each day she/he is in the neonatal unit?’) had a 97% positive response. This is an important aspect of parent care involvement, and we retained this question.
- Inter-item correlation. We assessed the questions with numerical answers (21/28) for interitem correlation. A strong correlation was seen between two sets of questions: B2 (‘Have you been given enough written information (in paper or electronic form) to help you understand your baby’s condition and treatment?’) and B12 written (‘On a scale of 1 to 10 how satisfied are you with how you receive information about your baby on the neonatal unit? In written information’) (correlation coefficient: −0.775, p<0.001), indicating that parents who felt they were given enough written information were likely to be more satisfied with the method of communication being ‘written information’. B12 verbal (‘On a scale of 1 to 10 how satisfied are you with how you receive information about your baby on the neonatal unit? In verbal updates’ and B12 overall ‘On a scale of 1 to 10 how satisfied are you with how you receive information about your baby on the neonatal unit overall? (correlation coefficient: −0.726, p<0.001), indicating that parents who were more satisfied with verbal updates were likely to be more satisfied with overall communication.

We retained all questions as they assessed different areas of care.

**Table 3:** Reformatting examples for survey questions with highest non-response rates

Question	Non-response rate (%)	Details of reformatting
B12 (four parts)	44	On a scale of 1–10, how satisfied are you with how you receive information about your baby on the neonatal unit? This question contains four subquestions (verbal information, telephone information, written information and overall). There was a large proportion of non-respondents on analysis for telephone (34%), written (44%) and overall (8.1%). As these performed well during cognitive testing, this was likely due to the questions’ format. We have added a letter prefix to each subquestion (a, b, c and d) so it is more obvious all questions need to be answered. We have added the response option N/A to all subquestions.
B14 open	73	If there is anything else you would like to tell us about how you receive information about your baby on the neonatal unit, then please do so here. In view of the high proportion of non-respondents and the fact that another open question at the end allows parents to give similar feedback, we removed this question.
E1 open	61	If there is anything else you would like to tell us about the way you are given updates about your baby on the neonatal unit, then please do so here. After removing question B14, this remains the survey’s only generic open question. We reworded it to ‘If there is anything else you would like to tell us about your experience of care on the neonatal unit then please do so here.’

N/A, not applicable.

### Refined PEC survey

The original Picker survey contained 61 questions in 10 sections, worded in the past tense and capturing a broad experience of neonatal care. The refined PEC questionnaire contains 28 questions in five sections (including Likert scale and free-text questions) to enable prospective evaluation and focus on the principles of family-centred care (online supplemental files 3 and 4)

10.1136/fetalneonatal-2022-324548.supp3 Supplementary data

## Discussion

We report the development, refinement and performance evaluation of a 28-question prospective parent experience survey, in collaboration with parents of babies in neonatal care, the PEC questionnaire. This refined parent experience survey, which focuses on communication and parent involvement, was evaluated to have favourable performance in the neonatal parent population, and can be used to assess parent experience in UK neonatal care. Inconsistency in and lack of validation of survey instruments measuring parent satisfaction in neonatal care (and specifically with family centred care) have been highlighted worldwide.^8 14^ The lead author’s systematic review of interventions aiming to improve parent experience of neonatal care identified that less than 20% of studies used fully validated surveys.^8^ Most surveys are administered around the time of discharge from neonatal care and retrospectively assess parent experience, like the recently developed CO-PARTNER tool, which measures parent participation and collaboration with staff.^19^ Among validated surveys, no existing survey in the UK measures real-time parent feedback in neonatal care. Therefore, the PEC survey is the first parent experience survey in the UK refined and formally evaluated for use while infants are still receiving neonatal care. Internationally, one real-time tool exists, developed since the PEC’s inception; the nine-question ‘digiFCC-P’.^20^ This administers one question a day to parents in neonatal care via text message, evaluating the quality of family-centred care. Comparison between the two tools and evaluation within the UK neonatal parent population would be important future work.

Strengths of this study include parent codesign with the parent representative throughout (maintaining the parent perspective) and the evaluation and feedback from parents of infants currently receiving NHS neonatal care. Containing 28 questions, PEC is substantially shorter than the original 61-question Picker survey. Ninety-eight per cent of parent respondents completed all questions until the penultimate question, and >50% of respondents dropped off at the last question. Non-responses to the last question were expected, as this was an open question.

A limitation was the recruitment of parents that could speak and understand written English, thereby potentially excluding parents from different cultures who may experience neonatal care differently. Because the self-administered survey was only available in English, revising and assessing the survey in other languages would require one-to-one interpreters and a cultural survey revision, which was not within this study’s remit.

Another limitation is that this study was undertaken at a single centre; however, the wide parent sample used for evaluation analysis included mothers and fathers, and a range of ethnicities and infants’ gestational age.

A sole researcher conducted cognitive interviews with parents; however, feedback was reviewed in revision cycles together with Picker methodology experts.

Due to the nature of the study, parents were recruited for cognitive testing by convenience sampling. We conducted a small number of parent cognitive interviews; however, this sample was deemed sufficient in view of extensive prior validation assessment of the original survey (27 cognitive interviews). As anticipated, our analysis reached data saturation within six interviews. Our parent sample included five mothers and one father, as more mothers were present on the neonatal unit during the day. While our interviews predominantly explored maternal views, the original Picker survey’s extensive cognitive testing included both mothers and fathers, as did our own PEC survey analysis.

The PEC survey is available under licence to NHS neonatal services, via Picker. It can be used as part of service evaluation on neonatal units and for continuous assessment of parent experience in research, audit and quality improvement projects. Its use will support the iterative development, piloting and improvement of family-centred interventions in neonatal care. As this is the first survey to be used for real-time feedback in neonatal care, it is intended that baseline per-question scores are established for each neonatal unit by initial survey administration, ahead of introducing any new intervention. Scores would be anticipated to differ between neonatal units, with the aim to achieve as close as possible to 100% for the parent satisfaction questions and to monitor the unit-specific scores for staff/parent interaction questions, as appropriate for each neonatal unit. Change in unit level scores would therefore be appropriate to use to monitor the impact of any intervention over time.

Future research could assess the updated survey to explore how it functions following minor refinements made, to evaluate the survey for use in other English-speaking countries, to investigate translation to other languages and to explore how parent responses vary in relation to changes in infants’ clinical status/outcomes alone.

## Conclusion

Adapted from the 2014 UK Parents’ Experience of Neonatal Care Picker survey, the PEC survey is the first in the UK and one of two tools in English-speaking countries worldwide, where formal evaluation supports its use in collecting real-time parent feedback in neonatal care. The PEC survey focuses on communication and parent involvement, enabling continuous assessment and iterative improvement of family-centred interventions in neonatal care, and is available under licence.

## Data Availability

Data are available upon reasonable request. An anonymised dataset of PEC surveys completed by parents was analysed for this study’s validation analysis.
