# Supplementary material for: Parents’ Experiences of Communication in Neonatal Care (PEC): a neonatal survey refined for real-time parent feedback
Source: Arch Dis Child Fetal Neonatal Ed. 2023 Jan 30;108(4):416–20. doi: 10.1136/archdischild-2022-324548 (PMC10314049; doi:10.1136/archdischild-2022-324548)
Supplement: Supplementary data [file fetalneonatal-2022-324548supp001.pdf]

Online supplementary file 1. PEC survey Steering Group

PEC survey Steering Group (10 members):

- Consultant Neonatologist (CG- author)
- Senior neonatal speciality trainee (SS- author)
- Parent with neonatal experience and study's parent representative (SK- author)
- Parent with neonatal experience
- Two senior neonatal nurses (one of which also neonatal research nurse)
- Bliss parental support neonatal charity representative
- Two methodology research experts from Picker (AT- author)
- Health services researcher and statistician from Picker (AJP- author)
