# Supplementary material for: Parents’ Experiences of Communication in Neonatal Care (PEC): a neonatal survey refined for real-time parent feedback
Source: Arch Dis Child Fetal Neonatal Ed. 2023 Jan 30;108(4):416–20. doi: 10.1136/archdischild-2022-324548 (PMC10314049; doi:10.1136/archdischild-2022-324548)
Supplement: Supplementary data [file fetalneonatal-2022-324548supp002.pdf]

Online supplementary file 2. New question added for more depth following parent interviews

Question: *'Have you been able to speak to a doctor about your baby as much as you want?'*

1. Yes, definitely;

2. Yes, to some extent

3. I have not wanted or needed to speak to a doctor

4. No

Most parents answered *'2. Yes to some extent'* during cognitive testing. Parents raised issues such as staff shortages on weekends and wanted the opportunity to say that.

Added a routing option to answers 2 and 4, redirecting parents to an additional question:

'What is the main reason you have not been able to speak to a doctor as much as you want?

1. Doctors don't appear approachable

2. Doctors appear too busy

3. Doctors are not present on the neonatal unit when I am there

4. I do not understand the doctors' explanations

5. Other (please specify)
