# Supplementary material for: Parents’ Experiences of Communication in Neonatal Care (PEC): a neonatal survey refined for real-time parent feedback
Source: Arch Dis Child Fetal Neonatal Ed. 2023 Jan 30;108(4):416–20. doi: 10.1136/archdischild-2022-324548 (PMC10314049; doi:10.1136/archdischild-2022-324548)
Supplement: Supplementary data [file fetalneonatal-2022-324548supp004.pdf]

The PEC questionnaire:

Parents' **E**xperiences of **C**ommunication in Neonatal care

What is the survey about?

This survey is about your **parent experience** in the neonatal unit currently providing care to your baby.

Completing the questionnaire

The word 'baby' is used throughout to refer to either a single baby or more than one baby.

The word 'staff' is used throughout to refer to both neonatal doctors and nurses.

The questionnaire should take around 10 minutes to complete. For most questions, please tick clearly inside one box ☑ using a black or blue pen. For some questions you may be asked to tick more than one box. For questions with a range of answers (1-10) please circle your chosen answer.

Not all sections will apply to you, and sometimes you will find the box you have ticked has an instruction to go to another question. By following the instructions carefully, you will only answer the questions that apply to you.

Please **do not** write your name or address anywhere on the questionnaire.

Taking part in this survey is voluntary. Your answers will be treated in confidence.

Thank you for completing this survey.

YOU AND YOUR BABY

This information will help us ensure we include the experiences of a diverse group of parents. All answers will be anonymised and kept confidential for use within our research team.

Please circle what applies:

What is your age?

- Under 18
- 18-24
- 25-29
- 30-34
- Over 35

What is your gender?

- Female
- Male
- Other (please specify) ________

What is your ethnic group?

- White
- Mixed
- Asian or Asian British
- Black or Black British
- Chinese
- Other (please specify) ________

At how many weeks gestation was your baby born?

____

How long has your baby been on this neonatal unit for?

- Under 1 week
- 1-2 weeks
- 2-4 weeks
- 1-2 months
- 2-4 months
- 4-6 months
- Over 6 months

Sakonidou S, *et al. Arch Dis Child Fetal Neonatal Ed* 2023; 108:416–420. doi: 10.1136/archdischild-2022-324548

SECTION A. STAFF ON THE NEONATAL UNIT

A1. Have the neonatal unit's **rules, procedures and facilities for parents** been clearly explained to you?

1 ☐ Yes, definitely
2 ☐ Yes, to some extent
3 ☐ No
4 ☐ Can't remember

A2. Has the purpose of the **machines, monitors and alarms** used in the neonatal unit been clearly explained to you?

1 ☐ Yes, definitely
2 ☐ Yes, to some extent
3 ☐ No
4 ☐ Don't know / can't remember

A3. Have infection control practices been explained to you, such as **hand washing and procedures for visitors?**

1 ☐ Yes, definitely
2 ☐ Yes, to some extent
3 ☐ No
4 ☐ Don't know / can't remember

A4. Have you been told which nurses are responsible for your baby's care **each day** she/he is in the neonatal unit?

1 ☐ Yes
2 ☐ No

A5. Have you been able to talk to staff on the unit about your worries and concerns?

1 ☐ Yes, always or nearly always
2 ☐ Yes, sometimes
3 ☐ No
4 ☐ I have had no worries or concerns

A6. Have you been able to speak to a doctor about your baby **as much as you want?**

1 ☐ Yes, definitely → **go to A8**
2 ☐ Yes, to some extent → **go to A7**
3 ☐ I have not wanted or needed to speak to a doctor → **go to A8**
4 ☐ No → **go to A7**

A7. What is **the main reason** you have **not** been able to speak to a doctor as much as you want?

1 ☐ Doctors don't appear approachable
2 ☐ Doctors appear too busy
3 ☐ Doctors are not present on the neonatal unit when I am there
4 ☐ I do not understand the doctors' explanations
5 ☐ Other (please specify): ________________

____

____

A8. Have staff given you conflicting information about your baby's condition or care?

1 ☐ Yes, often
2 ☐ Yes, sometimes
3 ☐ No, not at all

A9. Overall, do you have confidence and trust in the staff members that have been caring for your baby?

1 ☐ Yes, always or nearly always
2 ☐ Yes, sometimes
3 ☐ No

Sakonidou S, *et al. Arch Dis Child Fetal Neonatal Ed* 2023; 108:416–420. doi: 10.1136/archdischild-2022-324548

SECTION B. INFORMATION AND SUPPORT FOR PARENTS

B1. If you ask questions about your baby's condition and treatment, do you get answers you can understand?

1 ☐ Yes, always or nearly always
2 ☐ Yes, sometimes
3 ☐ No
4 ☐ I have not asked any questions

B2. Have you been given enough written information **(in paper or electronic form)** to help you understand your baby's condition and treatment?

1 ☐ I have not wanted or needed any written information
2 ☐ Yes, definitely
3 ☐ Yes, to some extent
4 ☐ No, I have not been given enough written information
5 ☐ No, I have not been given any written information

B3. Have you had an opportunity to go through *your baby's medical notes with staff in the neonatal unit? *(See box below)*

> *Your baby's medical notes:*
> *are **NOT the nursing notes** left with the baby on the cot.*
> *They are notes SECURELY STORED WITH STAFF and contain medical information about your baby (plans from ward rounds etc)*

1 ☐ I did not know about the medical notes, but had I known I would have wanted to
2 ☐ I did not know about the medical notes, but had I known I still wouldn't have wanted to
3 ☐ Yes
4 ☐ No, but I want to
5 ☐ No, but I do not want to

B4. In the last 24 hours, how many times have you telephoned the neonatal unit?

1 ☐ One time
2 ☐ Two times
3 ☐ Three or more times
4 ☐ I have not telephoned the unit in the last 24 hours → **go to B6**

B5. If you telephoned the unit, what was the reason for your call?

PLEASE SELECT ALL THAT APPLY

1 ☐ I wanted information about ward round times
2 ☐ I wanted information about visiting times
3 ☐ I wanted information about parking
4 ☐ I wanted a general update about my baby
5 ☐ I wanted to discuss a change in my baby's care
6 ☐ I wanted information about breastfeeding and/or expressing
7 ☐ I wanted to ask for test results (please specify what type of tests): ____________
8 ☐ I wanted to discuss a specific part of my baby's care (please specify which part):
____
____
9 ☐ Other (please specify): ______________
____

B6. In the last 24 hours, how many times have you asked your baby's **nurse** for an update about your baby, face-to-face?

1 ☐ One time
2 ☐ Two times
3 ☐ Three or more times
4 ☐ I have not asked my baby's nurse for an update face-to-face in the last 24 hours

B7. On a scale of 1 to 10 how satisfied are you with the **frequency of updates** you receive from **nurses** about your baby on the neonatal unit?

Extremely dissatisfied — Extremely satisfied

1 2 3 4 5 6 7 8 9 10

B8. When **ward rounds** take place, are you present when your baby is being discussed?

1 ☐ Yes, always or nearly always
2 ☐ Yes, sometimes
3 ☐ No, I have not been allowed to be there
4 ☐ No, the ward round times are not convenient for me
5 ☐ Other (Please specify)________________

____

B9. In the last 24 hours, how many times have you asked to speak with a **doctor (outside ward round times)**?

1 ☐ One time
2 ☐ Two times
3 ☐ Three or more times
4 ☐ I have not asked to speak with a doctor in the last 24 hours → **go to B11**

B10. If you asked to speak with a doctor, what did you want to discuss?

PLEASE SELECT ALL THAT APPLY

1 ☐ I wanted a general update about my baby
2 ☐ I wanted to discuss a change in my baby's care
3 ☐ I wanted to ask for test results (please specify which test): ________________
4 ☐ I wanted to discuss a specific part of my baby's care (please specify which part): ____________
5 ☐ Other (please specify): ______________

____

B11. On a scale of 1 to 10 how satisfied are you with the **frequency of updates** you receive from **doctors** about your baby on the neonatal unit?

Extremely dissatisfied — Extremely satisfied

1 2 3 4 5 6 7 8 9 10

B12. On a scale of 1 to 10 how satisfied are you with **how** you receive information about your baby on the neonatal unit?

	Extremely dissatisfied									Extremely satisfied	
**a. Verbal updates**	1	2	3	4	5	6	7	8	9	10	**N/A**
**b. Telephone calls**	1	2	3	4	5	6	7	8	9	10	**N/A**
**c. Written information**	1	2	3	4	5	6	7	8	9	10	**N/A**
**d. Overall**	1	2	3	4	5	6	7	8	9	10	**N/A**

B13. "I get the information I need to understand my baby's health status".

1 ☐ Strongly agree
2 ☐ Agree
3 ☐ Neither agree nor disagree
4 ☐ Disagree
5 ☐ Strongly disagree

Sakonidou S, *et al. Arch Dis Child Fetal Neonatal Ed* 2023; 108:416–420. doi: 10.1136/archdischild-2022-324548

SECTION C. YOUR INVOLVEMENT IN YOUR BABY'S CARE

C1. Do the neonatal unit staff members include you in discussions about your baby's care and treatment?

1 ☐ Yes, always
2 ☐ Yes, sometimes
3 ☐ No

C2. Have you been told about any **changes** in your baby's condition or care?

1 ☐ Yes, always or nearly always
2 ☐ Yes, sometimes
3 ☐ No, I was not told about changes
4 ☐ Not sure/can't remember

C3. Overall, do staff help you feel confident in **caring** for your baby? **(nappy changes, feeds etc.)**

1 ☐ Yes, definitely
2 ☐ Yes, to some extent
3 ☐ No

C4. Are you involved as much as you want in the day-to-day care of your baby?

1 ☐ Yes, definitely
2 ☐ Yes, to some extent
3 ☐ No, I am not involved as much as I want
4 ☐ No, my baby is too ill

SECTION D. LEAVING THE NEONATAL UNIT

D1. How likely are you to recommend this neonatal unit to friends and family, if their baby needed similar care or treatment?

1 ☐ Extremely likely
2 ☐ Likely
3 ☐ Neither likely nor unlikely
4 ☐ Unlikely
5 ☐ Extremely unlikely
6 ☐ Don't know

SECTION E. YOUR COMMENTS

E1. If there is anything else you would like to tell us about your experience of care on the neonatal unit then please do so here.

THANK YOU VERY MUCH FOR YOUR HELP
