# Supplementary material for: Parents’ Experiences of Communication in Neonatal Care (PEC): a neonatal survey refined for real-time parent feedback
Source: Arch Dis Child Fetal Neonatal Ed. 2023 Jan 30;108(4):416–20. doi: 10.1136/archdischild-2022-324548 (PMC10314049; doi:10.1136/archdischild-2022-324548)
Supplement: Supplementary data [file fetalneonatal-2022-324548supp003.pdf]

picker Institute Europe
Making patients' views count

Bliss
for babies born too soon, too small, too sick

NHS England

Parents' experiences of Neonatal Care

NOT FOR DISTRIBUTION

What is the survey about?

This survey is about your baby's care **in the neonatal unit named in the letter sent with this questionnaire**. This may have been a Special Care Baby Unit (SCBU), a High Dependency Unit (HDU) or a Neonatal Intensive Care Unit (NICU).

Unless otherwise stated, please answer all questions **about the neonatal unit named in the letter** that came with this questionnaire. If you have had more than one experience of a baby who was cared for on this neonatal unit, please only think about your **most recent experience** when answering these questions.

Your views are very important in helping us find out what parents think of neonatal services and how they can be improved.

Who should complete the questionnaire?

The questions should be answered by the parent(s) or guardian(s) named on the front of the envelope.

Completing the questionnaire

The word 'baby' is used throughout to refer to either a single baby or more than one baby.

The questionnaire should take around 20 minutes to complete. For most questions, please tick clearly inside one box ☑ using a black or blue pen. For some questions you may be asked to tick more than one box.

Not all sections will apply to you, and sometimes you will find the box you have ticked has an instruction to go to another question. By following the instructions carefully, you will only answer the questions that apply to you.

Please **do not** write your name or address anywhere on the questionnaire.

Taking part in this survey is voluntary. Your answers will be treated in confidence.

Questions or help?

If you have any questions, or if you would like to complete the questionnaire over the phone or with the help of an interpreter, please call **Freephone 0800 783 2896** and we will do our best to help. The line is open Monday to Friday 8am-8pm and on Saturdays from 9am-12pm.

NEO14

Sakonidou S, *et al. Arch Dis Child Fetal Neonatal Ed* 2023; 108:416–420. doi: 10.1136/archdischild-2022-324548

SECTION A. BEFORE YOUR BABY WAS BORN

A1. **Before** your baby was born (i.e. during pregnancy or labour), did you know that they might need care in a neonatal unit?

1 ☐ Yes ➔ **Go to A2**

2 ☐ No ➔ **Go to B1**

A2. Before your baby was born (i.e. during pregnancy or labour), did a member of staff from the **neonatal unit** talk to you about what to expect after the birth?

1 ☐ Yes, definitely

2 ☐ Yes, to some extent

3 ☐ No

4 ☐ Don't know / can't remember

SECTION B. YOUR BABY'S ADMISSION TO NEONATAL CARE

B1. Was your baby **first admitted** to the neonatal unit named in the letter sent with this questionnaire?

1 ☐ Yes ➔ **Go to B2**

2 ☐ No, my baby was first admitted to a neonatal unit at a different hospital ➔ **Go to C1**

B2. After you gave birth, were you offered a photograph of your baby?

1 ☐ Yes

2 ☐ No, but I would have liked this

3 ☐ No, but I took a photograph myself

4 ☐ I did not want a photograph taken

5 ☐ Don't know/ can't remember

B3. After you gave birth, were you ever cared for in the same ward as mothers who had their baby with them?

1 ☐ Yes, and this bothered me

2 ☐ Yes, but I did not mind

3 ☐ No, I stayed in a separate room/area

4 ☐ I was discharged from hospital

B4. After your baby was admitted to the neonatal unit, were you able to see your baby as soon as you wanted?

1 ☐ Yes

2 ☐ No

3 ☐ No, but this was not possible for medical reasons (baby and/or mother)

4 ☐ No, because we were in different hospitals

SECTION C. STAFF ON THE NEONATAL UNIT

Please answer the following questions about the <u>unit named in the letter</u> that came with this questionnaire.

C1. When you visited the unit, did the staff caring for your baby introduce themselves to you?

1 ☐ All of the staff introduced themselves

2 ☐ Some of the staff introduced themselves

3 ☐ Very few or none of the staff introduced themselves

4 ☐ Don't know / can't remember

C2. Were you given enough information about the neonatal unit (such as rules, procedures and facilities for parents)?

1 ☐ Yes, definitely

2 ☐ Yes, to some extent

3 ☐ No

4 ☐ Can't remember

C3. Was the purpose of the machines, monitors and alarms used in the neonatal unit clearly explained to you?

1 ☐ Yes, definitely

2 ☐ Yes, to some extent

3 ☐ No

4 ☐ Don't know / can't remember

NOT FOR DISTRIBUTION

C4. Were infection control practices explained to you, such as hand washing and procedures for visitors?

1 ☐ Yes, definitely
2 ☐ Yes, to some extent
3 ☐ No
4 ☐ Don't know / can't remember

C5. Were you told which nurses were responsible for your baby's care each day s/he was in the neonatal unit?

1 ☐ Yes
2 ☐ No

C6. Were you able to talk to staff on the unit about your worries and concerns?

1 ☐ Yes, always or nearly always
2 ☐ Yes, sometimes
3 ☐ No
4 ☐ I had no worries or concerns

C7. Were you able to speak to a doctor about your baby as much as you wanted?

1 ☐ Yes, definitely
2 ☐ Yes, to some extent
3 ☐ No
4 ☐ I did not want or need to speak to a doctor

C8. Were the **nurses** on the unit sensitive to your emotions and feelings?

1 ☐ Yes, always or nearly always
2 ☐ Yes, sometimes
3 ☐ No
4 ☐ This was not necessary

C9. Were the **doctors** on the unit sensitive to your emotions and feelings?

1 ☐ Yes, always or nearly always
2 ☐ Yes, sometimes
3 ☐ No
4 ☐ I had no contact with doctors on the unit
5 ☐ This was not necessary

C10. In your opinion, was important information about your baby passed on from one member of staff to another?

1 ☐ Yes, always or nearly always
2 ☐ Yes, sometimes
3 ☐ No, information was not passed on
4 ☐ Don't know/ can't remember

C11. Did staff give you conflicting information about your baby's condition or care?

1 ☐ Yes, often
2 ☐ Yes, sometimes
3 ☐ No, not at all

C12. Did staff refer to your baby by his/her first name?

1 ☐ Yes, always or nearly always
2 ☐ Yes, sometimes
3 ☐ No
4 ☐ My baby did not have a name

C13. Overall, did you have confidence and trust in the staff caring for your baby?

1 ☐ Yes, always or nearly always
2 ☐ Yes, sometimes
3 ☐ No

NOT FOR DISTRIBUTION

Sakonidou S, *et al. Arch Dis Child Fetal Neonatal Ed* 2023; 108:416–420. doi: 10.1136/archdischild-2022-324548

SECTION D. YOUR INVOLVEMENT IN YOUR BABY'S CARE

Still thinking about when you were in the neonatal unit named in the letter that came with this questionnaire…

D1. Were you involved as much as you wanted in the day-to-day care of your baby, such as nappy changing and feeding?

1 ☐ Yes, definitely
2 ☐ Yes, to some extent
3 ☐ No, I was not involved as much as I wanted
4 ☐ No, my baby was too ill

D2. Did you have as much skin-to-skin contact with your baby as you wanted?

1 ☐ Yes, definitely
2 ☐ Yes, to some extent
3 ☐ No, not as much skin-to-skin contact as I wanted
4 ☐ No, but this was not possible for medical reasons
5 ☐ I did not know about skin-to-skin contact

D3. Did the neonatal unit staff **include you in discussions** about your baby's care and treatment?

1 ☐ Yes, always
2 ☐ Yes, sometimes
3 ☐ No

D4. Were you told about any **changes** in your baby's condition or care?

1 ☐ Yes, always or nearly always
2 ☐ Yes, sometimes
3 ☐ No, I was not told about changes
4 ☐ Not sure/can't remember

D5. When a ward round was taking place, were you **allowed** to be present when your baby was being discussed?

1 ☐ Yes, always or nearly always
2 ☐ Yes, sometimes
3 ☐ No, I was not allowed to be there
4 ☐ Not sure / I did not know about ward rounds

D6. Where possible, did staff arrange your baby's care (such as weighing, bathing) to fit in with your usual visiting times?

1 ☐ Yes, always or nearly always
2 ☐ Yes, sometimes
3 ☐ No, but I would have liked this
4 ☐ No, but this was not necessary

D7. Overall, did staff help you feel confident in caring for your baby?

1 ☐ Yes, definitely
2 ☐ Yes, to some extent
3 ☐ No

Feeding your baby

D8. If you wanted to **express** breast milk for your baby, were you given the **support** you needed from neonatal staff?

1 ☐ Yes, definitely ➔ **Go to D9**
2 ☐ Yes, to some extent ➔ **Go to D9**
3 ☐ No ➔ **Go to D9**
4 ☐ I did not want to express milk ➔ **Go to D10**
5 ☐ I could not express milk ➔ **Go to D10**

D9. When you were in the neonatal unit, were you given the **feeding equipment** you needed for expressing, such as a breast pump and sterilisation equipment?

1 ☐ Yes, definitely
2 ☐ Yes, to some extent
3 ☐ No

NOT FOR DISTRIBUTION

D10. Were you given enough privacy in the neonatal unit for expressing milk and/or breastfeeding your baby?

1 ☐ Yes, definitely
2 ☐ Yes, to some extent
3 ☐ No and it bothered me
4 ☐ No, but I did not mind
5 ☐ This was not needed

D11. If you wanted to **breastfeed** your baby, were you given enough support to do this from neonatal staff?

1 ☐ Yes, definitely
2 ☐ Yes, to some extent
3 ☐ No
4 ☐ I did not want to breastfeed my baby
5 ☐ My baby was unable to breastfeed for medical reasons

D12. If you fed your baby **formula milk**, were you given enough support to do this from neonatal staff?

1 ☐ Yes, definitely
2 ☐ Yes, to some extent
3 ☐ No
4 ☐ I did not want to feed my baby formula milk

SECTION E. ENVIRONMENT AND FACILITIES

Still thinking about the neonatal unit in the hospital named in the letter…

E1. Were you given enough privacy when discussing your baby's care on the neonatal unit with staff?

1 ☐ Yes, always or nearly always
2 ☐ Yes, sometimes
3 ☐ No
4 ☐ No, but I did not mind

E2. Was there enough space for you to sit alongside your baby's cot in the unit?

1 ☐ Yes, definitely
2 ☐ Yes, to some extent
3 ☐ No

E3. In your opinion, was there adequate security on the neonatal unit?

1 ☐ Yes, definitely
2 ☐ Yes, to some extent
3 ☐ No

E4. If you wanted to stay overnight to be close to your baby, did the hospital offer you accommodation?

1 ☐ Yes, always or nearly always
2 ☐ Yes, sometimes
3 ☐ No
4 ☐ I did not want/need to stay overnight

E5. Were you able to visit your baby on the unit as much as you wanted to? *(please only think about unit-related reasons and not personal reasons such as needing to care for other children)*

1 ☐ Yes, definitely
2 ☐ Yes, to some extent
3 ☐ No
4 ☐ I did not visit my baby

SECTION F. INFORMATION AND SUPPORT FOR PARENTS

Still thinking about the neonatal unit in the hospital named in the letter…

F1. If you asked questions about your baby's condition and treatment, did you get answers you could understand?

1 ☐ Yes, always or nearly always
2 ☐ Yes, sometimes
3 ☐ No
4 ☐ I did not ask any questions

NOT FOR DISTRIBUTION

F2. Were you given enough **written information** to help you understand your baby's condition and treatment?

1 ☐ Yes, definitely

2 ☐ Yes, to some extent

3 ☐ No, I was not given enough written information

4 ☐ I did not get any written information

5 ☐ I did not want or need any written information

F3. Did you have an opportunity to go through your baby's **medical notes** (not just the nursing notes) with staff while they were in the neonatal unit?

Please note – medical notes are not the notes left with the baby on the cot, but are stored securely with staff

1 ☐ Yes

2 ☐ No, but I wanted to

3 ☐ No, but I did not want to

4 ☐ I did not know about the medical notes

F4. Were you offered emotional support or counselling services from neonatal unit staff?

1 ☐ Yes

2 ☐ No, but I would have liked to have been offered this

3 ☐ I did not need any emotional support or counselling

F5. Were you given **enough information** about help you could get with expenses related to your baby's stay in the neonatal unit (such as travelling/parking expenses, hardship funds or food vouchers)?

1 ☐ Yes, definitely

2 ☐ Yes, to some extent

3 ☐ No

4 ☐ I did not need/want this information

F6. Did **staff give** you any information about parent support groups, such as Bliss or other local groups? (TICK ONE ONLY)

1 ☐ Yes, **staff gave** me this information

2 ☐ No, but this information was available in **the unit** (e.g. a leaflet in the parents' room)

3 ☐ No, I did not get this information

4 ☐ Don't know / Can't remember

SECTION G. LEAVING THE NEONATAL UNIT

G1. In total, how long did your baby stay in **neonatal care** (include all the hospitals they stayed in)?

1 ☐ Up to 1 week

2 ☐ More than 1 week but less than 4 weeks

3 ☐ Between 4 and 8 weeks

4 ☐ More than 8 weeks, but less than 12 weeks

5 ☐ 12 weeks or more

G2. When your baby was discharged from the neonatal unit, where did they go next?

1 ☐ A hospital maternity ward ➔ **Go to G4**

2 ☐ Another ward in the hospital ➔ **Go to G4**

3 ☐ Home ➔ **Go to G3**

4 ☐ Somewhere else ➔ **Go to G4**

G3. Were you offered overnight accommodation with your baby at the hospital before they left the neonatal unit?

1 ☐ Yes

2 ☐ No, but I would have liked it

3 ☐ No, but I did not want or need it

NOT FOR DISTRIBUTION

Sakonidou S, *et al. Arch Dis Child Fetal Neonatal Ed* 2023; 108:416–420. doi: 10.1136/archdischild-2022-324548

G4. Did you feel prepared for your baby's discharge from neonatal care?

1 ☐ Yes, definitely

2 ☐ Yes, to some extent

3 ☐ No

G5. Were you given enough information on what to expect in terms of your baby's **progress** and **recovery**?

1 ☐ Yes, definitely

2 ☐ Yes, to some extent

3 ☐ No

G6. How likely are you to recommend this neonatal unit to friends and family, if their baby needed similar care or treatment?

1 ☐ Extremely likely

2 ☐ Likely

3 ☐ Neither likely nor unlikely

4 ☐ Unlikely

5 ☐ Extremely unlikely

6 ☐ Don't know

SECTION H. YOU AND YOUR BABY

H1. Did your baby stay in a neonatal unit in more than one hospital?

1 ☐ Yes ➔ **Go to H2**

2 ☐ No ➔ **Go to H3**

H2. Did your baby spend **most** of its time in the neonatal named in the letter that came with this questionnaire?

1 ☐ Yes, my baby spent most time in the unit named in the letter that came with this questionnaire

2 ☐ No, my baby spent most of its time in another unit

3 ☐ My baby stayed a similar amount of time in two or more different units

H3. Did you give birth to a single baby, twins or more in your most recent pregnancy?

1 ☐ A single baby

2 ☐ Twins

3 ☐ Triplets, quads or more

H4. Roughly how many weeks pregnant were you when your baby was born?

1 ☐ Before I was 25 weeks pregnant

2 ☐ When I was 25 to 29 weeks pregnant

3 ☐ When I was 30 to 32 weeks pregnant

4 ☐ When I was 33 to 37 weeks pregnant

5 ☐ When I was 38 weeks pregnant or more

H5. How much did your **baby weigh at birth**? (*If you had twins or more than two babies, please fill in this question about the baby who was born first*)

1 ☐ Less than 1000g (1kg / 2lb 3oz)

2 ☐ 1000g to 1500g (1kg to 1.5.kg / 2lb 3oz to 3lb 5oz)

3 ☐ 1500g to 2500g (1.5kg to 2.5kg / 3lb 5oz to 5lb 8oz)

4 ☐ More than 2500g (2.5kg / 5lb 8oz)

H6. How many babies have you given birth to before this pregnancy?

1 ☐ None ➔ **Go to H8**

2 ☐ 1-2 ➔ **Go to H7**

3 ☐ 3 or more ➔ **Go to H7**

H7. Have you previously had a baby admitted to a neonatal unit?

1 ☐ Yes

2 ☐ No

NOT FOR DISTRIBUTION

H8. Who filled in this questionnaire?

1 ☐ The baby's mother

2 ☐ The baby's father

3 ☐ Parents together

4 ☐ The baby's guardian(s)

H9. To which of these ethnic groups would you say the mother of the baby belongs? (Tick ONE only)

1 ☐ White British

2 ☐ Any other white background

3 ☐ Mixed

4 ☐ Asian or Asian British

5 ☐ Black or Black British

6 ☐ Any other ethnic group

H10. Do you have a long-standing physical or mental health problem or disability?

1 ☐ Yes ➔ **Go to H11**

2 ☐ No ➔ **Go to H12**

3 ☐ I'd rather not say ➔ **Go to H12**

H11. Does this problem or disability affect your day-to-day activities?

1 ☐ Yes, definitely

2 ☐ Yes, to some extent

3 ☐ No

H12. In what **year** was the mother of the baby born?

(Please write in) e.g. | 1 | 9 | 7 | 4 |

Y	Y	Y	Y

SECTION J. YOUR COMMENTS

If there is anything else you would like to tell us about the neonatal care received in the **hospital named in the letter** sent with the questionnaire, then please do so here:

THANK YOU VERY MUCH FOR YOUR HELP

Please post this questionnaire back in the FREEPOST envelope provided. No stamp is needed

This survey is supported by Bliss, the special care baby charity. If this survey has raised any issues for you please contact Bliss on their free family support helpline number: 0500 618140 or visit: www.bliss.org.uk [Charity number: 1002973]

NOT FOR DISTRIBUTION
